# Whole-Genome Analysis of Temporal Gene Expression during Early Transdifferentiation of Human Lung Alveolar Epithelial Type 2 Cells *In Vitro*

**DOI:** 10.1371/journal.pone.0093413

**Published:** 2014-04-01

**Authors:** Helena Morales Johansson, Donna R. Newman, Philip L. Sannes

**Affiliations:** Department of Molecular Biomedical Sciences, Center for Comparative Medicine and Translational Research, College of Veterinary Medicine, North Carolina State University, Raleigh, North Carolina, United States of America; University of Pittsburgh, United States of America

## Abstract

It is generally accepted that the surfactant-producing pulmonary alveolar epithelial type II (AT2) cell acts as the progenitor of the type I (AT1) cell, but the regulatory mechanisms involved in this relationship remain the subject of active investigation. While previous studies have established a number of specific markers that are expressed during transdifferentiation from AT2 to AT1 cells, we hypothesized that additional, previously unrecognized, signaling pathways and relevant cellular functions are transcriptionally regulated at early stages of AT2 transition. In this study, a discovery-based gene expression profile analysis was undertaken of freshly isolated human AT2 (hAT2) cells grown on extracellular matrix (ECM) substrata known to either support (type I collagen) or retard (Matrigel) the early transdifferentiation process into hAT1-like cells over the first three days. Cell type-specific expression patterns analyzed by Illumina Human HT-12 BeadChip yielded over 300 genes that were up- or down-regulated. Candidate genes significantly induced or down-regulated during hAT2 transition to hAT1-like cells compared to non-transitioning hAT2 cells were identified. Major functional groups were also recognized, including those of signaling and cytoskeletal proteins as well as genes of unknown function. Expression of established signatures of hAT2 and hAT1 cells, such as surfactant proteins, caveolin-1, and channels and transporters, was confirmed. Selected novel genes further validated by qRT-PCR, protein expression analysis, and/or cellular localization included SPOCK2, PLEKHO1, SPRED1, RAB11FIP1, PTRF/CAVIN-1 and RAP1GAP. These results further demonstrate the utility of genome-wide analysis to identify relevant, novel cell type-specific signatures of early ECM-regulated alveolar epithelial transdifferentiation processes in vitro.

## Introduction

Lung alveolar epithelial type I (AT1) cells, critical for O_2_/CO_2_ exchange and fluid transport, are very vulnerable to damage, and delayed regeneration or replacement of these cells leads to respiratory distress [1]–[3]. Furthermore, the turnover of cells in the lung is slow compared to other organs [4]. Although some questions remain about the source of the progenitor cell that replaces the damaged AT1 cell, there is strong evidence that adjacent type II (AT2) cells can undergo limited proliferation and transdifferentiation into AT1 cells [5]–[7]. Other mechanisms of repair include the possibility of stem cells originating from the blood as well as from distal lung to repair injured lung epithelium [8]–[10].

Characterization of fetal lung development and alveolarization has revealed the involvement of a variety of growth factors which include Fgf10, Wnt7b, and VEGF, as well as a number of key transcription factors [11]–[13]. Furthermore, VEGFR2 and FGFR1 are important in lung regeneration regulated by pulmonary capillary endothelial cells in response to a reduction in pulmonary function [14]. Several signatures for mature AT2 and AT1 function and maintenance have been identified, such as surfactant proteins for AT2 and AQP5 and caveolin-1 for AT1 cells [3], [15]–[19]. However, little is known about whole-genome gene expression changes triggered during the early stages of AT2 transition into AT1 cells. Attempts to perform whole-genome gene expression analysis to characterize AT2 and AT1 in depth using LCM techniques have proven difficult due to the technical issues surrounding the relevant cellular morphology. Instead, primary AT2 and AT1 cells have been isolated using alternative purification schemes [20]. Gene expression analysis has identified additional novel genes up-regulated in freshly isolated AT1 cells in comparison to AT2 cells [20], [21]. A similar analysis of whole-genome expression changes during hAT2 transdifferentiation has not been previously reported. Primary AT2 cells in culture undergo limited proliferation and rapidly and spontaneously transdifferentiate into an AT1-like cell over a period of 3 to 8 days [22], [23]. Freshly isolated AT2 cells lose the ability to express their specific markers, followed by the increased expression of AT1 signatures with time in culture [24]. Therefore an in vitro analysis is an established, useful tool to identify additional functional groups that are regulated during early stage transdifferentiation.

The purpose of this study was a discovery-based examination of the early transdifferentiation process. We sought to identify, by whole-genome gene expression analysis, novel gene candidates that might play roles in the early transition of freshly-isolated human lung alveolar type II cells into hAT1-like cells over a time course from the day of attachment (“day 0”) to three days afterward. To accomplish this, the known influence of extracellular matrix substrata to differentially modulate the transdifferentiation process was exploited [25]. Our analysis identified functional groups of genes that are differentially regulated in hAT2 cells cultured on type I collagen, which enhances the transdifferentiation process, compared to those maintained as hAT2 cells on Matrigel. Five new genes of interest that were found to be specifically regulated during early transdifferentiation included *PLEKHO1, SPRED1, SPOCK2, PTRF/CAVIN,* and *RAB11FIP1*, which were selected for validation and additional analysis.

## Materials and Methods

### Cell procurement

Human AT2 (hAT2) cells were isolated from organ donor lungs from human subjects are provided by the Tissue Procurement and Cell Culture Core of the Cystic Fibrosis/Pulmonary Research and Treatment Center (the Core) at the University of North Carolina at Chapel Hill (UNC). All human materials were handled per protocols approved by the UNC Institutional Committee on the Protection of the Rights of Human Subjects (IRB) and strict procedures were followed to ensure patient confidentiality. Organ donor lungs not suitable for transplantation but still useful for cell harvest were obtained through the National Disease Research Interchange (Philadelphia, PA). Age, sex, and ethnic background will not be considered when obtaining specimens, and are expected to reflect those of the U.S. population of general organ donors. Uniform consent is not practicable or feasible because donors were deceased (i.e., cadaveric organ donors). For these specimens, consent for research use of tissue was obtained from an authorized representative of the deceased by the organ procurement agency and has been deemed acceptable by the IRB. The waiver does not adversely affect the rights and welfare of the tissue donors because of procedures to insure subject anonymity. The use of anonymous cadaveric organ donor tissue is considered to be exempt from IRB review. The IRB Committee has confirmed this exempt status UNC IRB protocol # 03-CFC-614 “Specimen Collection for Cystic Fibrosis Research” covers tissue banking by the Core.

### Cell culture

Human AT2 (hAT2) cells were isolated from organ donor lungs obtained by the University of North Carolina Cystic Fibrosis/Pulmonary Research and Treatment Center Tissue Procurement and Cell Culture Core (Chapel Hill, NC) and specifically depleted of fibroblasts using procedures as described previously, with minor modifications [26]. All human specimens (de-identified) were handled under Institutional Review Board-approved protocols. A summary of donor demographics is available as Figure S1. Isolated hAT2s were maintained in low-glucose DMEM medium supplemented with 10% fetal bovine serum and containing a mixture of penicillin, streptomycin, and amphotericin B (Mediatech, Manassas, VA), and gentamicin. Cells were cultured at 37°C under an atmosphere of 92.5% air and 7.5% CO_2_ in tissue culture dishes pre-coated with type I collagen from rat tail (BD Biosciences, Franklin Lakes, NJ) at a density of 0.06 μg/mm^2^ or pre-coated with Matrigel (BD Biosciences), as described previously [27]. Standard Matrigel contains laminin (56%), type IV collagen (31%), and entactin (8%), as determined by the manufacturer.

### RNA extraction

Primary isolates of lungs from three age-matched donors were used for the analysis of mRNA expression by Illumina Human HT-12 Bead Chip, resulting in triplicate samples per each of four time points (upon cell attachment to substrate  =  “day 0” and days 1, 2, and 3) on each substrate. Cells on collagen were harvested with Trizol (Invitrogen, Carlsbad, CA) and stored at −80°C until all time points were completed. Cells grown on Matrigel-coated dishes were detached from the matrix using Cell Recovery Solution (BD Biosciences), as per manufacturer's instructions, and collected cells were lysed in Trizol. Total RNA was prepared using a hybrid protocol for Trizol and RNeasy mini kits (Qiagen, Valencia, CA), as follows. Cells were lysed with Trizol, chloroform was added and samples spun to separate RNA from DNA and protein. Residual DNA was removed by passing the RNA-containing layer through a genomic DNA elimination column (Qiagen). The resulting flow-through was mixed with EtOH to precipitate RNA. The RNA was collected in an RNeasy mini column and washed with RNeasy kit solutions. RNA was eluted with RNase-free water. Samples were sent to DHMRI (Kannapolis, NC) to be prepared for the BeadChip arrays. Before labeling, RNA samples were quantitated using an ND-1000 spectrophotometer (NanoDrop, Wilmington, DE) and RNA quality was evaluated using a 2100 Bioanalyzer (Agilent Technologies, Santa Clara, CA). Samples were required to have a minimum RIN of 6.5 for gene expression array analysis.

### Amplification, labeling and BeadChip hybridization of RNA samples

Total RNA was used as template to amplify and label cRNA using the Illumina TotalPrep RNA Amplification Kit (Ambion, Austin, TX) as per manufacturer's instructions. cRNA synthesis and Illumina BeadChip array probing were performed at the microarray facility at David H. Murdock Research Institute, Kannapolis, NC. The labeled probes were then mixed with hybridization reagents and hybridized overnight to the Human HT-12 V4.0 BeadChip arrays (Illumina, San Diego, CA), permitting analysis of over 47,000 transcripts (http://www.illumina.com/products/humanht_12_expression_beadchip_kits_v4.ilmn). Following washing and staining, the BeadChips were scanned with Illumina IScan to measure fluorescence intensity at each probe. Each “probe” is designed for an isoform of a specific gene product and consists of 12–40 identical beads randomly spread out over the surface of the array. The raw data images were imported into Illumina Genome Studio, which generated an average intensity of each probe for each sample.

### Data analysis and processing

Quality control on the Human HT-12 BeadChip arrays was performed as described by the manufacturer and resulted in the inclusion of all arrays in the analysis. The intensity of the signal corresponds to the quantity of the respective mRNA in the original sample. The complete microarray dataset is MIAME compliant and is available at Gene Expression Omnibus, a public functional genomics data repository supporting MIAME-compliant data submissions (accession number GSE40516 at: http://www.ncbi.nlm.nih.gov/geo/query/acc.cgi?acc=40516). Following quality inspection, probe sets with a signal near the background noise were termed absent. All present signals were subjected to average normalization. Data analysis of the filtered probe set can be described in two steps. First, differential gene expression was calculated between Matrigel and collagen samples for the cells within each time point. This was accomplished using the free software R program version 2.14.0 Copyright (C) 2011, The R Foundation for Statistical Computing. Fold-change and P-values for each probe set were calculated using a moderated t-statistic, with the variance estimate being adjusted by incorporating global variation measures for the complete set of probes on the array. The P-value data were then corrected for multiple hypotheses testing using the Benjamini and Hochberg method [28]. A≥2.5 fold-change difference in absolute gene expression values between AT2 cells cultured on collagen and those cultured on Matrigel, at each time point over the entire time course, was considered significant. In addition, adjusted P-values of <0.01 and <0.05 were used as criteria for defining two sets of genes for further analysis.

### Bioinformatic data processing

The information for all the candidate genes (biological process, molecular function, and cellular component) was compiled manually based on the following web resources: Pubmed – www.ncbi.nlm.nih.gov/pubmed; KEGG – www.genome.jp/kegg; GeneCards - www.gencards.org; Human protein atlas - http://www.proteinatlas.org [29], [30] and is presented in Tables S1 and S2.

### Quantitative RT-PCR

Cells from lungs of two non-age-matched donors were cultured on collagen or Matrigel, as previously. Trizol was used to lyse cells and total RNAs were prepared as described for BeadChip analysis. Total RNA was transcribed into cDNA using the High Capacity cDNA Reverse Transcription Kit (Applied Biosystems, Foster City, CA) according to the manufacturer's instructions. TaqMan Quantitative Real-Time Polymerase Chain Reaction (qRT-PCR) and statistical methods were performed as previously described, with minor modifications [26]. TaqMan Gene Expression Assays and TaqMan Gene Expression Master Mix (Applied Biosystems) were used in all qRT-PCR reactions, which were run on a MyiQ iCycler (BioRad, Hercules, CA). qRT-PCR was conducted for SP-C, SPOCK2, SPRED1, PTRF/CAVIN-1, PLEKHO1, RAB11FIP1 and RAP1GAP. TBP and GAPDH were used for normalization and for calculation of mean fold change. REST-MCS, based on a modified ΔΔC_T_ method, was used to relatively quantify levels of mRNA transcripts in samples from isolates grown on each substrate as they changed over time compared to the “day 0” reference value for that substrate [31], [32].

### Protein preparation and immunoblotting

Time course experiments using cells from lungs of two additional age-matched donors were terminated by removing the media, rinsing the dishes once with PBS, and lysing cells with RIPA buffer (Thermo Scientific, Rockford, IL) with addition of protease inhibitor cocktail (Complete EDTA mini, Roche Diagnostics Corporation, Indianapolis, IN), phosphatase inhibitors (PhosSTOP, Roche) and PMSF (Sigma-Aldrich, St. Louis, MO). For subsequent protein extraction, cell lysates in RIPA buffer were sonicated and then centrifuged to remove cell debris. The total protein in each supernatant was quantified by Pierce 660 nm Protein Assay (Thermo Scientific). Equal amounts of protein from each sample were combined with Laemmli SDS dye containing 2-mercaptoethanol, heated, and electrophoretically separated on NuPAGE gels (Invitrogen) under reducing conditions, followed by transfer to nitrocellulose membranes. After blocking, the blots were probed overnight with primary antibodies: mouse anti-PTRF (Abcam, Cambridge, MA), rabbit anti-Rap1GAP (Abcam), rabbit anti-SP-D (Santa Cruz Biotechnologies, Santa Cruz, CA), mouse anti-caveolin-1 (BD Transduction Laboratories, San Jose, CA), and mouse anti-β-actin (Santa Cruz Biotechnologies). Specific bands were detected with HRP-conjugated secondary antibodies (Cell Signaling Technology, Danvers, MA) followed by chemiluminescence using Pierce SuperSignal West Pico or West Dura (Pierce, Rockford, IL) and visualized by autoradiography.

### Immunofluorescence

Freshly isolated hAT2 cells from lungs of two non-age-matched donors were allowed to attach to Lab-Tek II CC2 glass chamber slides (Thermo-Fisher, Rochester, NY) pre-coated with rat tail collagen (BD Biosciences). After allowing cells to attach overnight, non-viable cells and debris were removed by a medium change; thereafter, media were changed every second day. Cells were fixed with paraformaldehyde (Electron Microscopy Sciences, Hatfield, PA) and blocked with 3% BSA (Sigma, St Louis, MO) and 0.1% saponin (Calbiochem, San Diego, CA) in PBS, followed by overnight incubation with primary antibodies: rabbit anti-PTRF (Abcam), mouse anti-caveolin-1 (BD Transduction Laboratories), and goat anti-SP-C (Santa Cruz Biotechnologies). Bound primary antibody was visualized with AlexaFluor 594-labeled donkey anti-rabbit or donkey anti-mouse IgG antibody (Molecular Probes, Life Technologies) and coverslips were mounted with Prolong Gold Antifade with DAPI (Life Technologies). Fluorescence photomicrographs were taken on a Meiji MX6300 fluorescence microscope with an Infinity 3 camera.

Paraffin-embedded human lung tissue was prepared by cutting 1 cm^3^ pieces of tissue and immersing them in neutral buffered formalin (NBF) under moderate vacuum for one hour to remove air. After overnight fixation, tissue was transferred to 70% EtOH in preparation for paraffin-embedding and tissue sectioning. For immunofluorescence of paraffin-embedded tissue, cut sections on glass slides were deparaffinized with 4 changes of xylene and rehydrated with an EtOH series. The sections were then washed, blocked, and incubated with primary antibodies overnight at 4°C and processed as described above for cell monolayers on glass chamber slides.

## Results

### Major morphological and signature expression changes occur before day 2

**Figure 1:**
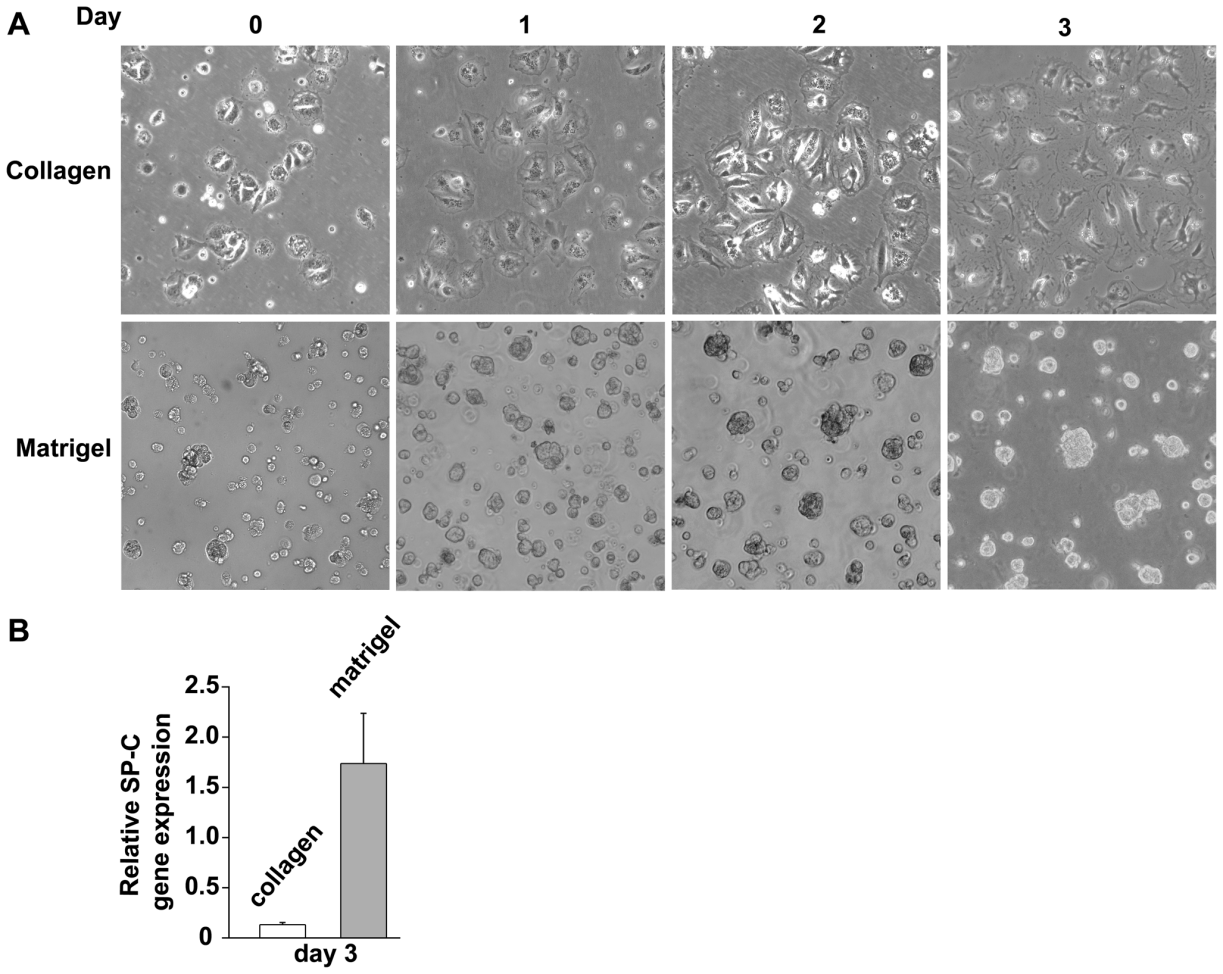
*In vitro* culture of adult human alveolar epithelial cells. (A) Human lung AT2 cell morphology changes more when cultured on rat-tail collagen-coated dishes than on Matrigel-coated dishes. Freshly-isolated human AT2 cells were seeded on collagen-coated or Matrigel-coated tissue culture dishes and photomicrographs were acquired after 12 hours (“Day 0”), 1, 2, and 3 days. Cells on collagen flattened and spread; cells on Matrigel remained cuboidal in shape and accumulated into enlarging “cysts”. Original magnification, 100X. (B) Gene expression analysis of Day 3 cells shows that SP-C, a marker of AT2 cells, is not expressed in cells cultured on collagen; however, its expression is retained on Matrigel.

Rat AT2 cells undergoing spontaneous differentiation into AT1-like cells in vitro show morphological changes and decreased expression of AT2 signatures, including loss of surfactant-containing lipid droplets, along with increased expression of AT1 signatures, such developing a more squamous appearance [15], [16]. Rat AT2 cells cultured on Matrigel tend to maintain AT2 signatures such as surfactant production for up to 10 days in culture [33], which make it a useful reference for non-transdifferentiating AT2 cells. In the present study, to examine the early timeframe for differentiation-related changes in vitro, freshly isolated hAT2 cells were seeded onto type-I collagen-coated dishes to induce transdifferentiation into hAT1-like cells or onto Matrigel to slow or retard it (see Materials and Methods). hAT2 cells seeded on collagen-coated dishes attached after 12 h and appeared AT2-like, cuboidal in shape, and with multiple lipid droplets (lamellar bodies) (Fig. 1A, top). Between day 0 and day 1, these cells spread and their phospholipid content became less evident as the cells spread and assumed a more squamous appearance (Fig. 1A, top). With time, the cell spreading increased and reached a maximum between day 4 and day 6, with nucleus and thin cytoplasmic ridges at the core surrounded by thinner extended cytoplasm (Fig. 1A, top). Thereafter, the AT1-like appearance was retained. Cells seeded on Matrigel attached within 12 h, retained a cuboidal appearance, and did not spread, as has previously been described [27] (Fig. 1A, bottom). hAT2 cells cultured on Matrigel formed small, clustered groups of various sizes within 2 to 4 days and retained this phenotype (Fig.1A, bottom). To evaluate AT2 signature surfactant protein-C (SP-C) expression in cells grown on the different substrates, freshly isolated hAT2 cells were cultured on collagen or Matrigel and harvested after 3 days. qRT-PCR analysis confirmed a significantly higher SP-C mRNA level in hAT2 cells on Matrigel than on collagen (Fig. 1B).

These results are in agreement with previous studies [33] which showed that the major morphological changes of individual transdifferentiating hAT2 cells in vitro occur between day 0 and day 3 after isolation and that the major changes in cells on Matrigel did not involve significant alterations in cellular morphology. Furthermore, the reduced gene expression of the hAT2 signature SP-C in hAT2 cells on collagen is consistent with transdifferentiation.

### Differential gene expression profiles of hAT2 cells on collagen versus Matrigel

To identify novel gene expression changes during the early transition to AT1-like cells, transdifferentiating (collagen) and non-transdifferentiating (Matrigel) hAT2 cells were harvested upon attachment (about 12 h after seeding to each matrix) and on each subsequent day, through day 3. Total RNA was isolated and transcribed into cRNA, which was then hybridized onto Illumina Human HT-12 BeadChips containing 46,000 probes to characterize whole genome gene expression. The analysis was set to identify genes with expression differences of ≥2.5 fold between the transitioning and non-transitioning AT2 cells. The analysis yielded 323 genes (after removing repeated probes for the same genes) displaying statistically significant differences between the substrates in their expression as they changed over time. Of these, there were 98 genes with a P value <0.01 (Table S1) and 225 genes with a P value <0.05 and >0.01 (Table S2). Genes expressed significantly differently over time in transdifferentiating AT2 cells compared to AT2 cells maintained on Matrigel were assigned to a specific functional group based on bioinformatics analysis (see Materials and Methods), as summarized in Figure S2. Major groups of genes have functions in signaling, the cytoskeleton, transcriptional regulation, cell growth regulation, immune system, transporters/channels, metabolic pathways, lipid metabolism, and extracellular components. There was also a large group of genes with unknown functions and a group of pseudogenes with no known protein products (Fig. S2). The distribution of significant genes among the 13 functional groups speaks to the functional importance of the influence of substrata, with signaling and cytoskeleton/cell structure functions predominating over the other groups in the total number and high significance of the affected genes (Fig. S2).

**Figure 2:**
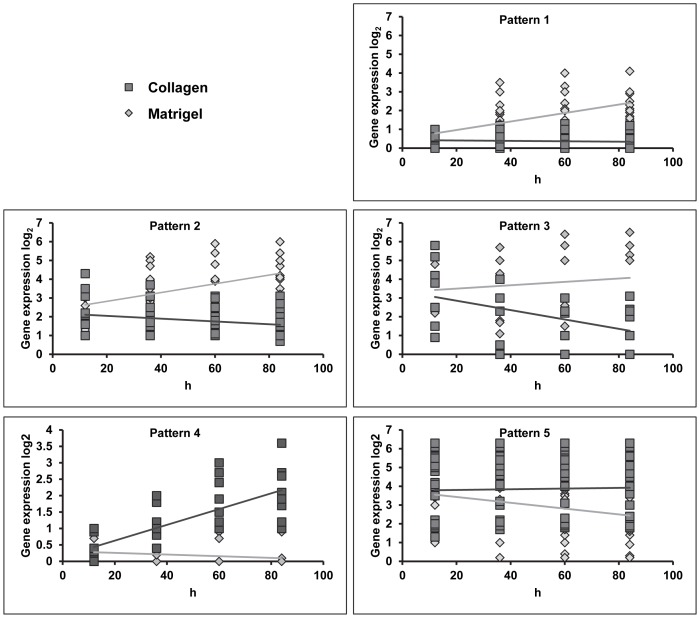
Candidate genes' expression patterns. Genes expressed differentially in hAT2 cells on collagen compared to Matrigel with P<0.01 were analyzed based on expression dynamics and sorted into one of five expression patterns. Gene expression data were graphed in Microsoft Excel as scatterplot graphs and means of expression were drawn which illustrate the patterns. Patterns 1, 2, and 3 include genes that are more highly expressed in cells on Matrigel than on collagen. Pattern 1 is characteristic of genes with low expression on both substrates at day 0 that increases only on Matrigel over time. Patterns 2 and 3 are characteristic of genes expressed at high levels on both substrates at day 0 and either increase on Matrigel while remaining stable (Pattern 2) or decrease (Pattern 3) in cells on collagen. Note that due to quantitative differences in baseline expression, Pattern 3 genes do not cluster at a particular y-axis value but do exhibit similar dynamics over their time courses. Patterns 4 and 5 include genes with higher expression in cells on collagen than on Matrigel. Pattern 4 genes exhibit very low expression on both substrates at Day 0 (12 hours after attachment) which increases steadily with time on collagen, while expression on Matrigel remains very low. Pattern 5 genes start at a moderate or high level of expression on both substrates that decreases over time only in cells on Matrigel. Note that due to quantitative differences in baseline expression, Pattern 5 genes do not cluster at a particular y-axis value but do exhibit similar dynamics over their time courses.

Further analysis of the gene expression data identified five different expression patterns (Fig. 2) among the highly significant 98 genes of Table S1. Three patterns, 1, 2 and 3, showed higher expression in hAT2 cells maintained on Matrigel compared to transdifferentiating hAT2 cells on collagen. In pattern 1, expression of genes in cells on both substrates began low; in cells on Matrigel, expression of these genes increased over time, while they remained low in cells on collagen. Patterns 2 and 3 showed high expression at day 0 but stable or decreasing expression, respectively, in transdifferentiating hAT2 cells. Two patterns, patterns 4 and 5, showed higher expression (increasing or stable, respectively) in transitioning hAT2 cells. Note that patterns 1 and 4 started near zero, with pattern 1 showing steady increases in expression on Matrigel and pattern 4 showing steady increases on collagen.

The candidate genes were then analyzed based on previously established expression and/or immunohistochemical localization data, as summarized in Tables S1 and S2, to identify genes not previously characterized in transdifferentiating AT2 cells. Known hAT1 signatures identified in this analysis were caveolin-1 [34], cytokeratins 6 and 7 [35], and connexin 43 [36], all with expression pattern 4 or 5. Known hAT2 signatures identified here were surfactant proteins [37], LAMC2 [38], calcium channels [39], CFTR [40], and fatty acid binding proteins [41], all with expression pattern 1, 2 or 3 (Fig. 2).

It would be logical that among those candidate genes with expression patterns 4 and 5 there should be some, not previously characterized, with roles in the morphology or function of AT1-like cells. Furthermore, as pattern 4 is characteristic of genes that are primarily expressed in cells grown on collagen, there may be some genes among those with this pattern that have a role in the transdifferentiation process itself.

### Validation of a subset of gene candidates by TaqMan qRT-PCR

Five genes, PLEKHO1, SPRED1, SPOCK2, PTRF/CAVIN and RAB11FIP1, were selected based on their putative function in hAT2 cells in signaling and extracellular matrix and their differential expression showing either pattern 4 or 5 (Fig. 2 and Table S1). To corroborate the differential expression of candidate genes in transitioning hAT2 cells grown on collagen as shown by Illumina BeadChip array, genes selected for further characterization were analyzed by qRT-PCR. For this purpose, hAT2 cells from three new isolations were cultured on collagen or Matrigel and harvested on days 0, 1, 2 and 3, as before.

**Figure 3:**
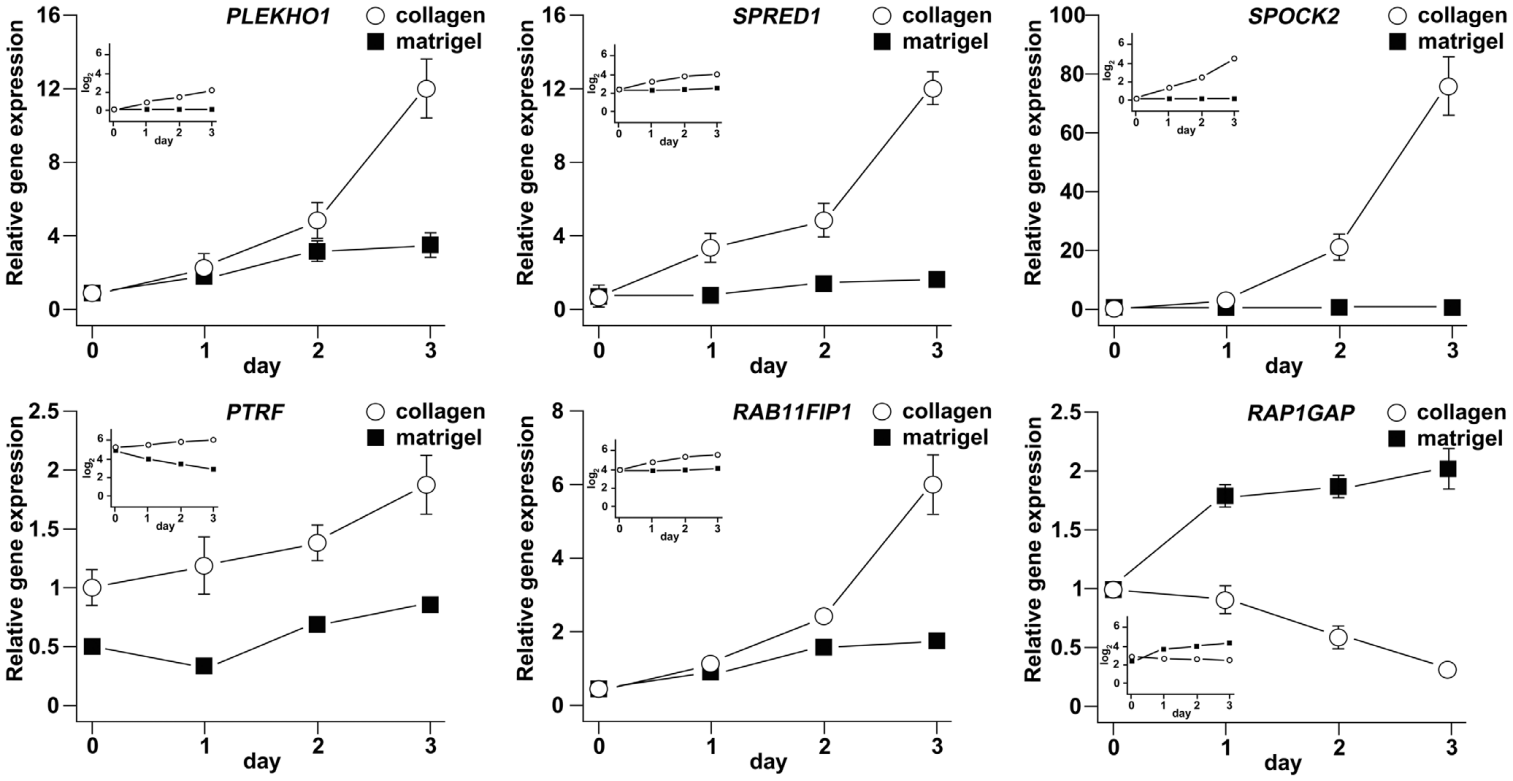
Validation of a subset of candidate genes by qRT-PCR. RNA was harvested from hAT2 cells cultured on collagen or Matrigel at 12 = 2. The relative expressions of SPOCK2, PLEKHO1, SPRED1, RAB11FIP1, PTRF/CAVIN-1, and RAP1GAP were assessed by TaqMan qRT-PCR and normalized to GAPDH and TBP. The fold-changes in relative expression over time are shown as graphs. The smaller, inset graphs show the expression results obtained from the Illumina BeadChip analysis, for comparison.

TaqMan assays gave raw cycle threshold (C_T_) numbers for the selected genes that indicated that each was at least moderately well-expressed throughout the time course. Gene expression analysis (ΔΔC_T_) confirmed that SPOCK2, PLEKHO1, SPRED1, RAB11FIP1, and PTRF/CAVIN-1 were up-regulated over time on collagen (Fig. 3), corroborating the Illumina BeadChip results and lending support to the idea that these genes may be reflective of or actively involved at an early stage in the in vitro transdifferentiation of hAT2 to hAT1-like cells.

Another gene, RAP1GAP, was selected for similar analysis based on the role of Rap1 signaling in control of permeability and cadherin-mediated attachment in epithelium and its up-regulation in non-transitioning AT2 cells with expression pattern 2 (Fig. 2 and Table S1). RAP1GAP expression was confirmed to be down-regulated on collagen and up-regulated on Matrigel over time (Fig. 3), corroborating the Illumina BeadChip results.

### Changes in protein expression and localization of the Caveolin-1 regulator PTRF/CAVIN-1 in transitioning hAT2 cells

**Figure 4:**
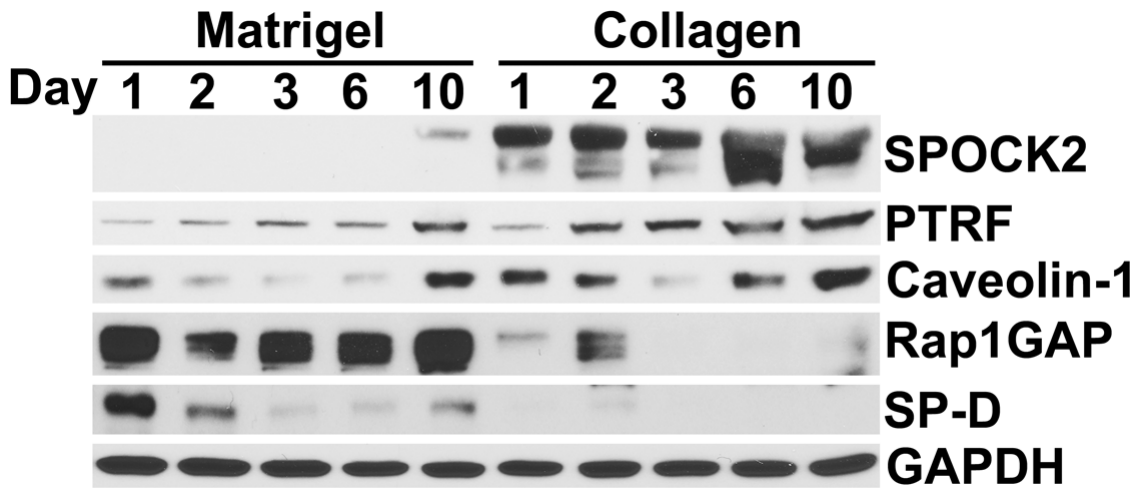
Time course protein expression analysis of selected candidate genes. SPOCK2 and PTRF/CAVIN-1 proteins increase in transitioning hAT2 cells on collagen over 10 days, while Rap1GAP is expressed mainly in AT2 cells on Matrigel. AT1 signature caveolin-1 and AT2 signature SP-D are shown to confirm phenotype. Freshly-isolated hAT2 cells were seeded on rat-tail collagen or Matrigel; cells were harvested for proteins on days 1, 2, 3, 6 and 10 after seeding. Results shown are representative of three separate time course experiments using cells from two isolations.

Based on the confirmation by qRT-PCR of the BeadChip expression results, regulation of selected candidate genes was further characterized at the protein expression level. For this purpose, total proteins were isolated from hAT2 cells cultured on collagen or Matrigel and harvested on days 1, 2, 3, 6 and 10. SP-D was detectable by Western blot in cells on Matrigel, whereas caveolin-1 was observed at higher levels on collagen throughout the time course, confirming each phenotype (Fig. 4). SPOCK2 and PTRF/CAVIN-1 were detectable at all time-points in the cells cultured on collagen. Rap1GAP was highly expressed at all time-points in cells on Matrigel but not in those on collagen, except for a weak increase on day 2 (Fig. 4). Despite relatively high levels of gene expression, as evidenced by relatively low raw C_T_ numbers at most time points, neither SPRED1 nor RAB11FIP1 was detected in any of the samples with available antibodies (data not shown).

**Figure 5:**
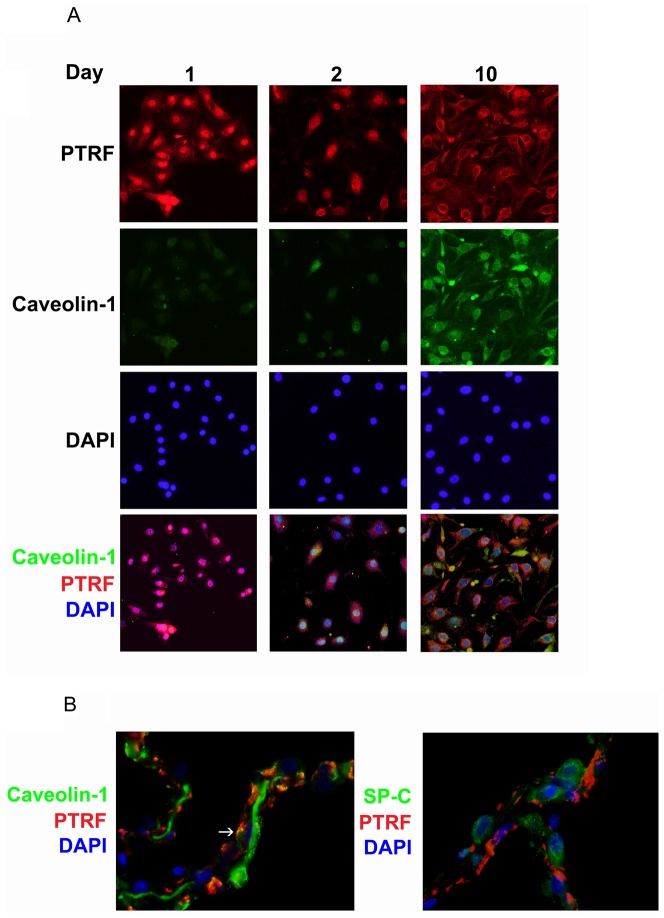
Sub-cellular localization of PTRF/CAVIN-1 changes from nuclear to cytoplasmic in transitioning hAT2 cells. (A) Cells from two separate isolations were seeded on collagen-coated glass chamber slides and fixed on days 1, 2, and 10. PTRF/CAVIN-1 (red) and caveolin-1 (green) were detected by immunofluorescence; DAPI (nuclei, blue) was supplied in the mounting medium. Nuclear PTRF (day 1) becomes cytoplasmic and co-localizes with caveolin-1 by day 10. Results shown are representative. Original magnification 40X. (B) Paraffin-embedded normal human lung tissue sections were deparaffinized and immunofluorescence was performed for PTRF/CAVIN-1 (red) and caveolin-1 (green, left panel) or SP-C (green, right panel); DAPI (nuclei, blue) was supplied in the mounting medium. Caveolin and PTRF co-localize while PTRF and SP-C do not. Results shown are representative of three separate cellular preparations. Original magnification 100X.

Further analysis of PTRF/CAVIN-1 by immunofluorescence revealed nuclear localization at day 1 (Fig. 5). At day 2, PTRF/CAVIN-1 localized to the nuclear periphery and later (day 10) appeared both at the nuclear periphery and in the cytoplasm. These results show that PTRF/CAVIN-1 protein expression increases in transitioning AT2 cells along with changes in its subcellular localization from nuclear to cytoplasmic, a process that begins between day 1 and day 2. Caveolin-1 fluorescence at day 1 and day 2 was weak. At day 10, the caveolin-1 signal was stronger and appeared cytoplasmic and similar to PTRF/CAVIN-1 in localization (Fig. 5). In normal human lung tissue, PTRF appeared to be highly expressed and co-localized with a subgroup of caveolin-1-staining areas, but not with SP-C (Fig. 5).

These results confirm the expression of PTRF in transitioning hAT2 cells and that of Rap1GAP in hAT2 cells. Also, the confirmation of PTRF co-localization with caveolin-1, both in transitioning hAT2 cells in vitro and in normal human lung tissue, lends validity to our use of the isolated hAT2 cell cultured on collagen as a model for the differentiated hAT1 cell.

## Discussion

In this discovery-based study, changes to the global mRNA transcriptome in adult human primary lung alveolar epithelial type 2 cells (hAT2 cells) cultured on collagen-coated surfaces to promote the rapid transdifferentiation into AT1-like cells [22] were compared to those in hAT2 cultured on Matrigel, which retards or otherwise slows this response [25], in order to identify new signatures of this process. The different matrix substrata promote phenotypic outcomes *in vitro* that should signify early shifts in gene expression. Interpretation of our results must take into account that this approach could unintentionally identify genes specifically responsive to extracellular matrix alone and which may be different from those responsive to other factors, such as growth factors or cytokines. Furthermore, differences between the complex composition of substrata actually encountered by hAT2 cells in a healthy whole lung and the simplified model substrata used here could lead to differences in non-physiological responses that must be appreciated when interpreting the data. Despite the limitations, the resultant data provide potentially important new perspectives on responsive gene groups and identify specific genes that may prove relevant to alveolar epithelial transdifferentiation.

Previous studies have focused on specific gene expression comparisons between primary rat AT2 or AT1 cells isolated by immunoselection with RTII70 or T1α (or RTI40) antibody [20], whereas this study sought to define early changes over the short transition period from isolation of primary human hAT2 cells to three days after their attachment to matrix. AT2 cells cultured on Matrigel have been extensively analyzed as maintaining AT2-specific signatures [22], [25], [42]–[45]. Accordingly, AT2 cells remain cuboidal on Matrigel whereas on collagen-coated dishes, cells spread out, gradually lose their numerous refractile lamellar bodies, and within 7 days appear morphologically similar to AT1 cells [46].

A previous study showing numerous differences in gene expression between freshly isolated rat AT1 cells and monolayers from primary rat AT2 cells cultured for 7 days to an “AT1-like” cell phenotype has led to an assumption that AT2 cells “de-differentiate” in vitro rather than transition into AT1 cells [20]. However, Fujino, et al., recently reported a FACS-based method for separating multiple viable cell populations from the adult human distal lung [47] using EpCAM, T1α, and VE-cadherin antibodies; immunofluorescence of the EpCAM^+^/T1α– subpopulation revealed this fraction to be nearly pure hAT2 cells, with 94% of cells expressing pro-SP-C. That the FACS-isolated hAT2 sub-population, cultured on collagen 1-coated slides for IF, continued to express pro-SP-C on Day 2 after seeding (the equivalent of our Day 1) and by Day 7 was expressing both AQP5 and T1α suggests that collagen 1 promotes transdifferentiation. The timing of this transition (after the equivalent of our Day 1) clearly supports our use of time points between attachment and Day 3 (cell spreading and disappearance of lamellar bodies being well underway) to investigate gene expression during transdifferentiation. Undoubtedly, the many alveolar microenvironment factors influencing characteristic gene expression are necessarily lost over the culture period, and there is currently no way to fully reconstitute that environment *in vitro*. Nevertheless, some signatures for AT1 cell function, such as caveolin-1, are observed to increase in AT2 cells with time in culture on collagen.

Despite the fact that the in vitro culture system cannot completely replicate the complete alveolar microenvironment, several lines of evidence support its use as a model for many physiological processes, such as surfactant production and ion transport [48], [49]. Many candidate genes from our study, such as ANXA2, LPLUNC1, CCL2, LAMC2, TUBB and FGFBP1, have been identified previously by other groups as active in lung alveolar epithelial cells. In this study, more than 150 genes are shown to have significantly increased mRNA levels in response to collagen compared to Matrigel, enabling the identification of genes that could play a role in the hAT2 transition into a hAT1-like cell. Interestingly, genes expressing known AT2 and AT1 signatures, such as several of the surfactant proteins (e.g. SFTPA, SFTPD) and caveolin-1, showed detectable changes in early expression (Tables S1 and S2), suggesting that subtle changes in expression do not necessarily rule out/diminish the presumptive importance of their respective role(s) in cell function. Of the four major surfactant genes, only SP-A and SP-D, but not SP-B or SP-C, were more highly expressed on Matrigel than on collagen, according to the Illumina BeadChip analysis. This was surprising since similar samples, analyzed by TaqMan qRT-PCR, revealed higher levels of SP-C in cells on Matrigel compared to collagen. A possible reason for this discrepancy could be the probe specificity. For instance, Affymetrix microarrays used to analyze gene expression of freshly isolated rat alveolar cells failed to show SP-C as more highly expressed in AT2 cells compared to AT1 cells [20]. Whereas the authors did find significant fold-change differences in SP-A and SP-B expression in their microarray analysis, upon qRT-PCR analysis the same genes showed much greater fold-change differences [20]. The apparent discrepancy was attributed, at least in part, to the insufficient specificity of short probes, but it was also acknowledged that signal saturation of probes for highly-expressed genes in the compared samples was a real possibility.

It may be important to note that the expression of certain genes and gene groups may be affected by techniques of cell isolation. In isolating hAT2 cells for the present study, significant attention was directed at the systematic depletion of fibroblasts [26], which can contaminate AT2 isolation procedures. The importance of the relationship *in vivo* between alveolar epithelial cells and the underlying fibroblast is well established and should not be underestimated [50], [51]. The fibroblasts remaining in many fresh AT2 preparations, which are often described as >95% pure, appear to have a significant impact on selected gene expression levels which increases with time in culture (unpublished observations). The fibroblast depletion procedure [26] used in the present study results in a near-absence of fibroblasts in the final hAT2 preparation and minimizes fibroblast contamination as a limiting factor in interpreting the results obtained. However, because the integrated crosstalk between the alveolar cell and the fibroblast *in vivo* may be necessary for the expression of certain phenotypic markers, our depletion protocol may alter the expression of established markers, based as they may be on *in vivo* expression patterns.

Although it was not within the scope of this study, changes in the expression of genes important for AT2 identity and, perhaps, regulators of the commitment to transdifferentiate may also be expected. This study identified as candidates some genes not previously linked to lung alveolar epithelial cell function, such as RAP2A, RAP1GAP, RAPGEF5, RAP1GDS1, and EPH4, previously identified as components of the Rap signaling pathway [52], [53]; this suggests the simultaneous expression of a functionally-related cluster of genes upon transdifferentiation (Tables S1 and 2). Rap2A and its paralog Rap1A are Ras-like small GTPases involved in control of permeability and cadherin-mediated attachment in epithelium [54]. Rap1GAP was selected for further examination due to its known role in regulating Rap signaling. We were not able to confirm the localization of Rap1GAP by immunofluorescence. However, Rap1GAP2, a protein closely related to Rap1GAP, has been localized to AT2 cells by immunohistochemistry, supporting a role for Rap signaling in lung epithelial cells [29], [53].

Although the expression changes are not as striking as those of the other candidate genes similarly verified by qRT-PCR, the cellular localization of PTRF/CAVIN-1 changed dramatically from nuclear to cytoplasmic in the early time points of transdifferentiation. PTRF/CAVIN-1 is nuclear as well in young fibroblasts but becomes cytoplasmic during cellular senescence [55]. This suggests that PTRF might play a dual role: as a transcription factor in the nucleus of non-transitioning hAT2 cells and then, during transdifferentiation and upon relocation to the cytoplasm, as being involved in caveolin-1 function. Targeted mutations in PTRF/CAVIN-1 result in lack of caveolae in lung epithelium and, along with increases in insulin and free fatty acid levels, PTRF mutant mice show impaired glucose tolerance and insulin resistance [56], [57]. On the cellular level, insulin (a known regulator of the PI3K and mTOR pathways) both changes the localization of PTRF and decreases its expression [58], [59], suggesting that the insulin pathway is important for PTRF function. In addition, insulin has been shown to play a role in lung epithelial fluid transport [55], [60], whereas caveolin-1 is a negative regulator of epithelial sodium channel (ENaC) function [61], which would be reflective of the AT2 to AT1 cell transdifferentiation process. In further support of this suggestion, cholesterol-chelating agents, which disrupt caveolin-1 translocation, and siRNA knockdown of the caveolin-1 gene both increase SP-C gene expression, while adenovirally-induced overexpression of caveolin-1 reduces SP-C gene expression [62].

Extracellular signaling via growth factors can be regulated by the extracellular matrix (ECM), which is composed of a complex mixture of sulfated proteoglycans that regulate the release of growth factors [63]. A previous study from our group has shown that the ECM at the basal surface of the AT1 cell is more sulfated than that of the AT2 cell, suggesting a role for ECM (and/or sulfate) in cellular maintenance [64]. We identified SPOCK2 as dramatically expressed both at the gene and the protein levels, suggesting a novel heparan sulfate proteoglycan (HSPG) component for the ECM of transdifferentiating hAT2 cells. A recent study revealed a role for SPOCK2/Testican-2 in bronchopulmonary dysplasia and alveolarization, which suggests a possible link between this HSPG and the transitioning of AT2 into AT1 cells [65], [66]. SPOCK2 expression increases significantly during alveolarization in rat lung development, with highest expression at Postnatal Day 18 (at termination of alveolarization) [65]. In addition, immunohistochemical experiments confirmed the expression of SPOCK2 during lung development, and the protein was found to be expressed throughout the extracellular matrix.

In this study, we attempted to identify candidate genes that could play role(s) in the transitioning of AT2 to AT1-like cells as modeled in vitro using different matrix substrata. Ideally, an in vivo approach would be preferable, but attempts using laser-capture microdissection (LCM) to isolate AT2 and AT1 cells from lung tissue have proven to be very difficult due to the significant loss of mRNA [20]. The discovery-based approach used here to study non-transitioning vs. transitioning human AT2 cells in vitro at multiple early time points provides insights into the dynamic shifts in expression of novel genes as well as confirmation of already established ones. Although we make no claim as to whether the reported changes in gene expression trigger, drive, or reflect transition, further studies of the physiological processes and pathways identified by whole genome gene expression analysis can increase our understanding of both broad and specific processes involved in AT2 to AT1 cell transition.

## Supporting Information

Figure S1 **Demographic data of lung donors.**
(TIF)

Figure S2 **Functional groups of the significant genes.** Genes differentially-regulated during the hAT2 to hAT1-like transition (>2.5-fold expression difference over the time course) identified by BeadChip analysis were assigned to a functional group based on bioinformatics analysis (see Materials and Methods). Results are presented in a bar graph comparing the number of differentially-regulated genes in each functional group, in which those bars labeled “C” represent the number of genes up-regulated on collagen in comparison to Matrigel, and those labeled “M” represent the number of genes up-regulated on Matrigel in comparison to collagen. Bar color denotes significant differences based on *p*<0.05 (light grey) or *p*<0.01 (dark grey).
(TIF)

Table S1 **Genes regulated by culture on collagen (C) or Matrigel (M) with high significance (p<0.01) over the entire time course.** Column headings: Exp prof lit, Expression profiling literature mentions or describes the gene in lung; Lung lit, identified in literature as linked to a function and/or expression in lung; Genecard Lung exp mRNA, from Genecard – Expression in lung noted: (+) weak, + moderate, ++ strong; IHC (HPA) AT1/AT2, Immunohistochemistry – from Human Protein Atlas – pneumocyte staining and in which cell type. Abbreviations: C, expression significantly higher on collagen substrate; M, expression significantly higher on Matrigel substrate; Y, yes; N, no; ?, tests were ambiguous; (AT1), immunohistochemistry identified protein in AT1 cells; (AT2), immunohistochemistry identified protein in AT2 cells.
(XLSX)

Table S2 **Additional genes regulated by culture on collagen (C) or Matrigel (M) with significance (p<0.05) over the entire time course.** Column headings: Exp prof lit, Expression profiling literature mentions or describes the gene in lung; Lung lit, identified in literature as linked to a function and/or expression in lung; Genecard Lung exp mRNA, from Genecard – Expression in lung noted: (+) weak, + moderate, ++ strong; IHC (HPA) AT1/AT2, Immunohistochemistry – from Human Protein Atlas – pneumocyte staining and in which cell type. Abbreviations: C, expression significantly higher on collagen substrate; M, expression significantly higher on Matrigel substrate; Y, yes; N, no; ?, tests were ambiguous; (AT1), immunohistochemistry identified protein in AT1 cells; (AT2), immunohistochemistry identified protein in AT2 cells.
(XLSX)
