# Supplementary figures and images for: Whole-Genome Analysis of Temporal Gene Expression during Early Transdifferentiation of Human Lung Alveolar Epithelial Type 2 Cells In Vitro
Source: PLoS One. 2014 Apr 1;9(4):e93413. doi: 10.1371/journal.pone.0093413 (PMC3972118; doi:10.1371/journal.pone.0093413)

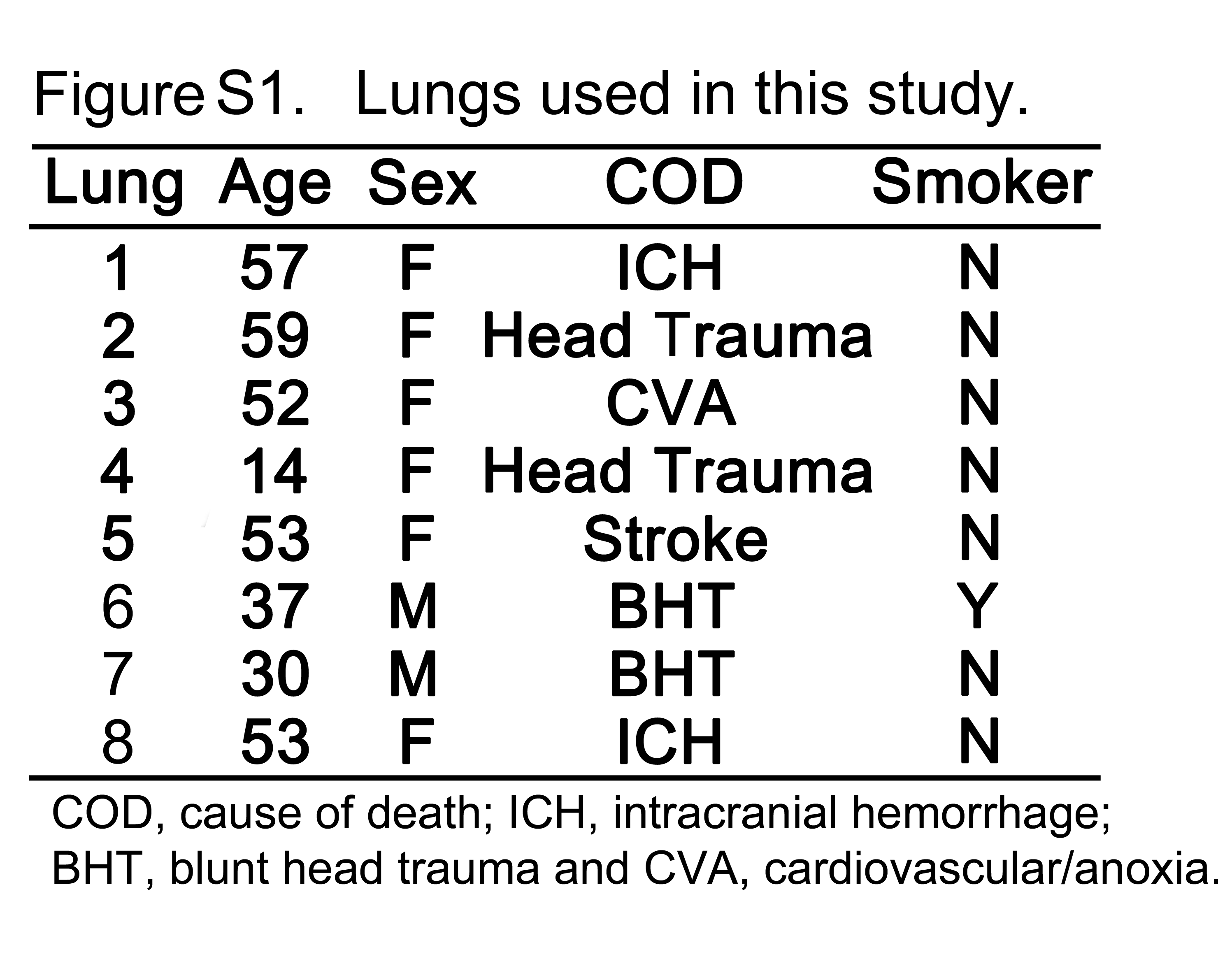

Supplement: Figure S1 — Demographic data of lung donors. (TIF) [file pone.0093413.s001.tif]

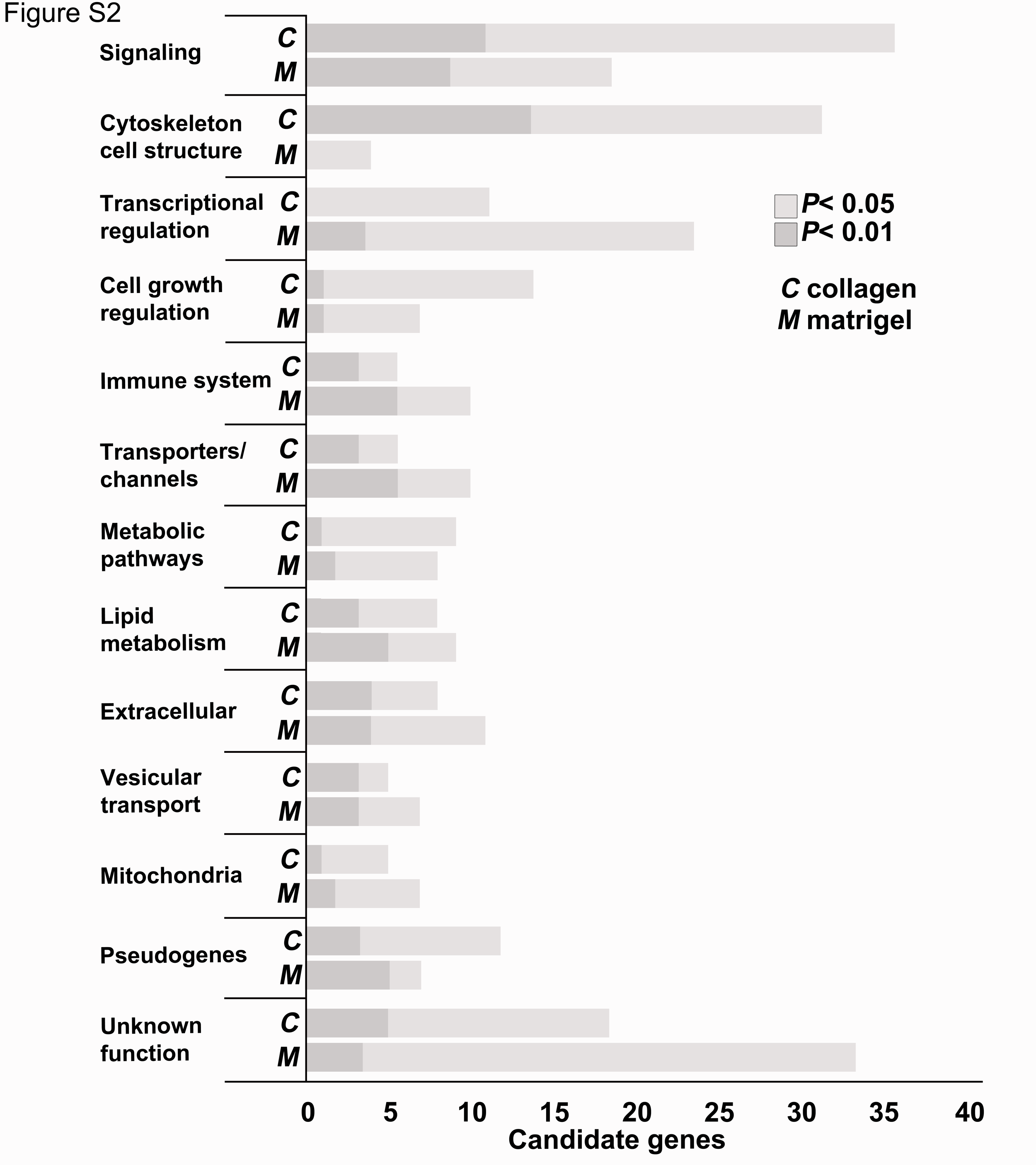

Supplement: Figure S2 — Functional groups of the significant genes. Genes differentially-regulated during the hAT2 to hAT1-like transition (>2.5-fold expression difference over the time course) identified by BeadChip analysis were assigned to a functional group based on bioinformatics analysis (see Materials and Methods). Results are presented in a bar graph comparing the number of differentially-regulated genes in each functional group, in which those bars labeled “C” represent the number of genes up-regulated on collagen in comparison to Matrigel, and those labeled “M” represent the number of genes up-regulated on Matrigel in comparison to collagen. Bar color denotes significant differences based on p<0.05 (light grey) or p<0.01 (dark grey). (TIF) [file pone.0093413.s002.tif]
